# Recovery Strategy for Overturned Wheeled Vehicle Using a Mobile Robot and Experimental Validation

**DOI:** 10.3390/s22165952

**Published:** 2022-08-09

**Authors:** Hidetoshi Ikeda, Shinya Atoji, Manami Amemiya, Shingo Tajima, Takayoshi Kitada, Kotaro Fukai, Keisuke Sato

**Affiliations:** 1 Department of Engineering, Niigata Institute of Technology, 1719 Fujihashi, Kashiwazaki City 945-1195, Japan; 2 Department of Mechanical Engineering, Toyama College, National Institute of Technology, 13 Hongouchou, Toyama 939-8630, Japan; 3 Department of Electrical and Control Systems Engineering, Toyama College, National Institute of Technology, Toyama 939-8045, Japan

**Keywords:** recovery, tipping over backward, recovery robot, AR marker, overturned vehicle

## Abstract

This paper describes mobile robot tactics for recovering a wheeled vehicle that has overturned. If such a vehicle were to tip over backward off its wheels and be unable to recover itself, especially in areas where it is difficult for humans to enter and work, overall work efficiency could decline significantly, not only because the vehicle is not able to perform its job, but because it becomes an obstacle to other work. Herein, the authors propose a robot-based recovery method that can be used to recover such overturned vehicles, and the authors evaluate its effectiveness. The recovery robot, which uses a mounted manipulator and hand to recover the overturned vehicle, is also equipped with a camera and a personal computer (PC). The ARToolKit software package installed on the PC detects AR markers attached to the overturned vehicle and uses the information they provide to orient itself in order to perform recovery operations. A statics analysis indicates the feasibility of the proposed method. To facilitate these operations, it is also necessary to know the distance between the robotic hand and the target position for grasping of vehicle. Therefore, a theoretical analysis is conducted, and a control system based on the results is implemented. The experimental results obtained in this study demonstrate the effectiveness of the proposed system.

## 1. Introduction

A number of developing countries are grappling with the serious twin problems of declining birthrates and aging populations, both of which reduce the total available workforce. In response to this, various parties have posited that if robots could be employed in dangerous areas and/or perform jobs that most people would not want to do because such work makes them feel mentally and physically distressed, the situation could be improved. Since many such jobs would require robots to move independently, various locomotive designs such as wheeled and tracked vehicles [1], special wheels [2], or the fusion of different mechanisms [3,4], have been widely researched. However, even though a great deal of effort has been directed at improving the movement capabilities of mobile robots, none of the methods investigated thus far are perfect. Each method has its merits and demerits, and every movement mechanism has its limitations.

Wheel-based mechanisms are currently the most widely researched for producing mobile robots. However, the primary problem with such mechanisms is their propensity to fall over or suffer from drive wheel slippage when it is necessary to navigate across rough terrain, run over obstacles, or when collisions occur with people or other robots. Overturning is especially problematic for wheeled robotic vehicles because such mishaps can result in fatal damage to the robot body. Hence, in cases where a robotic vehicle has overturned, the robot must be able to right itself, or it must be recovered in some other way. If this is not done, not only will the vehicle be unable to perform its assigned duties, but the vehicle itself becomes an obstacle that can prevent the free movement of humans or other robots. If such accidents occur in areas where access limitations make it difficult for people or other robots to enter and work, the overall workspace efficiency will decline significantly. Thus, it is clear that work efficiency would be significantly improved in areas where multiple robots exist and work together if they were designed with the ability to assist each other when mishaps such as overturning occur.

Some research programs have already been conducted to investigate scenarios involving obstacle avoidance and the recovery of overturned humanoid robots [5,6,7]. Other studies have examined ways to minimize damage when humanoid robots overturn [8], and a method of returning an overturned robot to a standing position has been studied [9]. For example, Tam and Kottege studied a fall avoidance and recovery method using a humanoid robot equipped with walking sticks [10].

In the present paper, the authors describe the design and construction of a robot system that is capable of recovering a robotic vehicle that has overturned and placing it back onto its wheels. To accomplish this, the recovery robot must be able to raise the overturned robotic vehicle while continuously controlling its inclination. However, when the mass of the overturned robotic vehicle is higher than that of the assisting robot, such a degree of control is difficult to achieve. That point was examined by Yoshida et al., and by Inaba et al., who studied pivoting transportation methods using a humanoid robot [11,12]. In a separate study, Asama et al. examined a transportation method using multiple robots that could raise an object [13].

In our previous studies, our research group investigated a step climbing method that is capable of inclining a robotic vehicle [14] or a handcart [15] in order to permit it to ascend a step.

The remainder of this paper is organized as follows. Section 2 describes our newly designed robot recovery system, while Section 3 describes the process of recovering a robotic vehicle that has fallen over backward. A statics analysis for a vehicle recovery is described in Section 4, Section 5 shows the results of our theoretical analysis, while Section 6 describes experimental results. Finally, Section 7 contains the conclusions.

## 2. Robot-Based Recovery System

**Figure 1:**
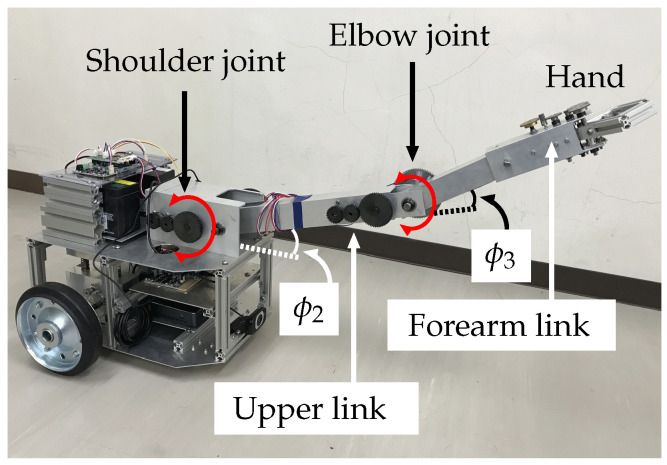
Recovery robot.

**Figure 2:**
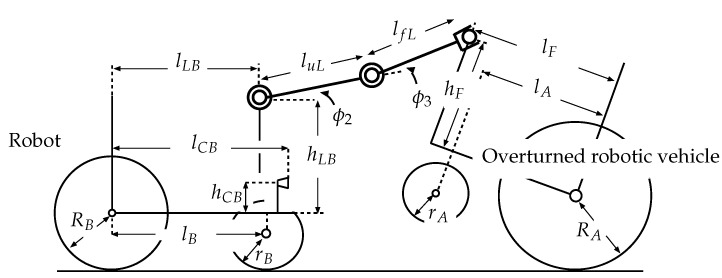
Model of robot and overturned robotic vehicle.

The wheeled recovery robot developed by our research group, which will be referred to as the “robot” hereafter, is shown in Figure 1. Table A1 in the Appendix A lists its specifications. Figure 2 shows a model of the robot and an overturned robotic vehicle. This robot has a manipulator that has two degrees of freedom (2DOF) and an attached hand that has 1DOF, for a total of 3DOF. It is mounted on the center of the robot’s body. Encoders are set on each axle of the robot manipulator and on the hand mechanism. In this paper, the length of the upper arm link (from the shoulder joint to the elbow joint) is $l_{uL}$, and the length of the forearm link (from the elbow joint to the hand) is $l_{fL}$. The angle of the shoulder joint is −30° $\leq \phi_{2}\leq$ +30°, and the angle of the elbow joint is −90° $\leq \phi_{3}\leq$ +90°.

**Figure 3:**
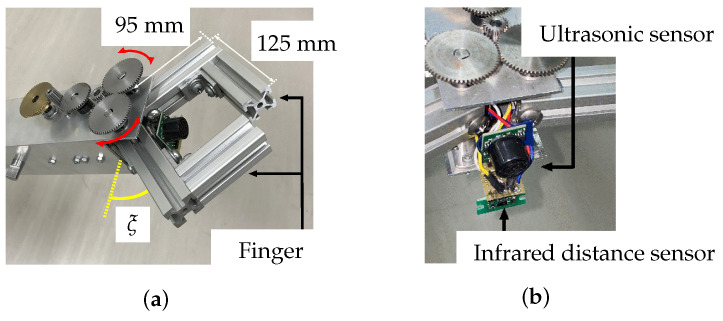
Robot hand mechanism: (**a**) fingers, (**b**) hand-mounted sensors.

The robot hand is equipped with two fingers, and the angle between them is 0° ≤ξ≤ +50°. (see Figure 3a.) The hand is equipped with an ultrasonic sensor (Digilent Pmod MAXSONAR) and an infrared sensor (POLORU VL6180X) for distance measurements, as shown in Figure 3b. A switch sensor (OMRON, D2F-L) is installed inside the hand mechanism to obtain contact information when the fingers are closed.

**Figure 4:**
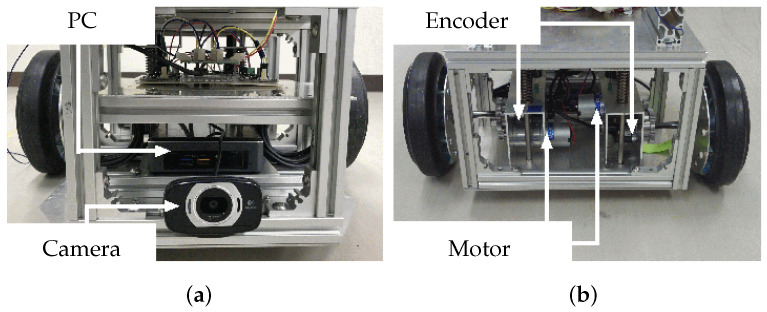
Views of (**a**) front and (**b**) rear of robot.

This robot has two pairs of left and right wheels mounted in tandem. The front pair are casters, and the rear pair are individually mounted driving wheels. The robot has motors and encoders on both driving wheel mechanisms that control its movements, as shown in Figure 4.

**Figure 5:**
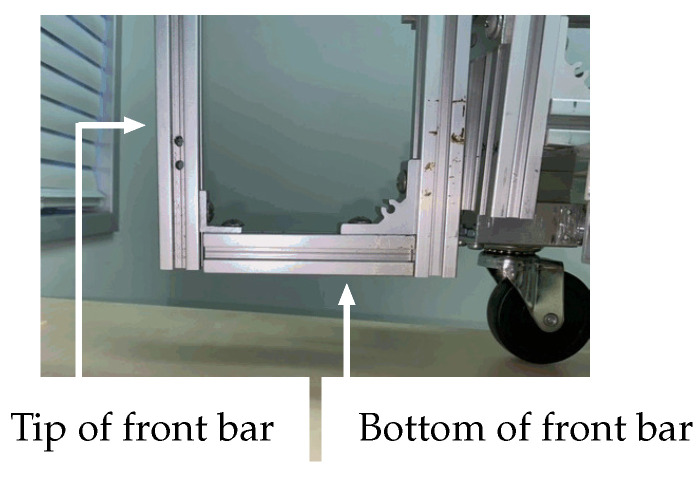
Front bar of vehicle.

**Figure 6:**
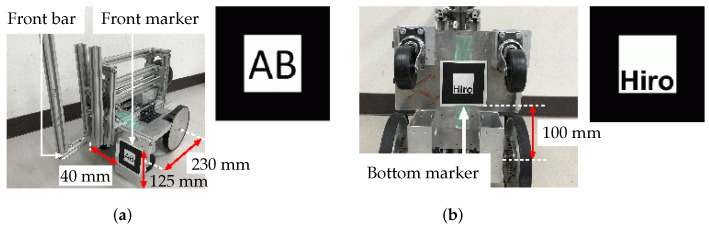
AR markers mounted on robotic vehicle: (**a**) front and (**b**) bottom.

When the robot approaches the overturned robotic vehicle, the manipulator grasps the front bar of the vehicle (Figure 5), using its hand mechanism (Figure 2 and Figure 3a) and attempts to help restore the overturned vehicle to its correct working position. Table A2 in the Appendix A lists the specifications of the overturned robotic vehicle, which is referred to as the “vehicle” hereafter in this paper (Figure 6). Like the recovery robot, the vehicle also has a wheeled mechanism that consists of front and rear wheel pairs. The front pair are casters, and the rear pair are individually mounted driving wheels.

**Figure 7:**
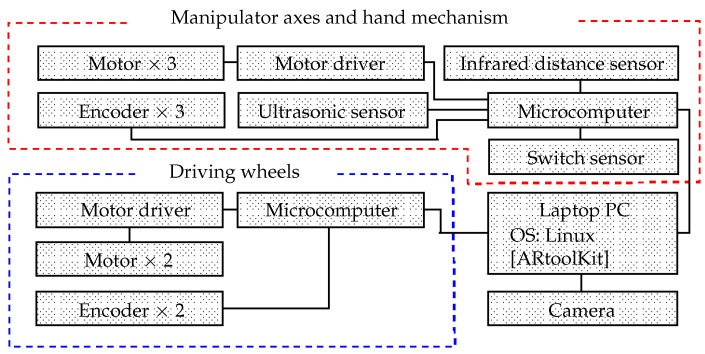
System configuration.

Figure 7 shows the system controls. The robot PC is equipped with an Intel (R) Core (TM) i3-7100U 2.40 GHz central processing unit (CPU) and 8 GB of random-access memory (RAM). The Linux (Lubuntu 18.04 LTS) operating system (OS) is installed. A Logicool HD Webcam C615 (angle of view: 74°) is mounted on the front of the robot and connected to the PC. The ARtoolkit software package is installed on the PC.

ARtoolKit is a software package that is capable of processing posture data and calculating the distance between the camera and an AR marker [16,17,18]. ARtoolkit users are given free permission to make and use AR markers in their systems, providing that they obey the rules governing their manufacture. However, before using ARtoolkit, users must register the AR markers used in their systems.

In this study, AR markers are mounted on the front (Figure 6a, Size: 70 mm × 70 mm) and bottom (Figure 6b, Size: 80 mm × 80 mm) of the overturned vehicle. The robot is able to obtain posture and distance information from the overturned vehicle based on data from these AR markers.

The robot is equipped with microcomputers (Arduino Holding, Arduino Leonardo) connected to the PC via Universal Serial Bus (USB) cables and connectors. Three motor driver circuits (Cytron Co., Ltd., MD10C) are connected to each microcomputer to control the driving processes. Electric motors (driving wheels: Tsukasa Electric Co., Ltd., TG-85E-SU-114-KA; shoulder and elbow axles: Tsukasa Electric Co., Ltd., TG-85E-KU-113-KA; and hand: HG16-240-AB-00) are connected to the motor driver circuits. Rotary encoders permit the degrees of these motors to be determined (driving wheels: Autonics, E30S4-100-3-N-5, shoulder axle, elbow axle, and hand mechanism: Taiwan Alpha Electronic, RE640F-40E3-20A-24P).

## 3. Overturned Vehicle Recovery Process

Initially, to recover a vehicle that has fallen off its wheels, the robot approaches the overturned vehicle and grasps it using the hand mechanism attached to its manipulator arm. In this paper, it is assumed that the camera on the robot is able to observe the AR markers on the overturned vehicle and that both the robot and vehicle are on a flat surface.

**Figure 8:**
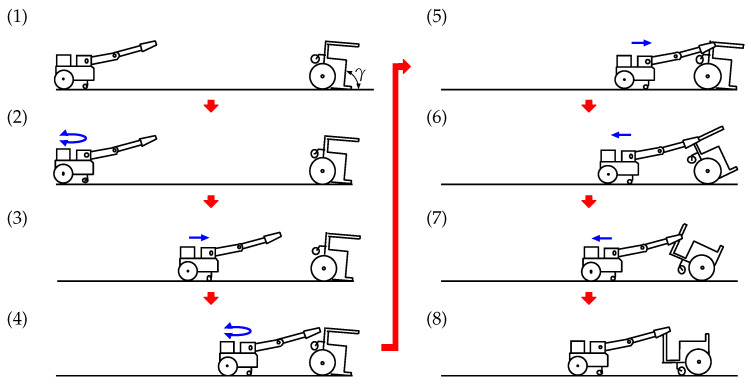
Overturned vehicle recovery process.

The recovery process is described in detail below, and each stage of the process is shown in Figure 8. The states shown in the figure correspond to (1)–(8) below:

(1) At this point, the vehicle has fallen over backward its inclination angle is γ. The camera on the robot captures an image of the overturned vehicle. ARtoolKit identifies the AR marker mounted on the bottom of the vehicle body and calculates the distance between the robot and the vehicle, as shown in Figure 6b.
(2) The robot drives its left and right wheels to pivot around its center position until it is oriented towards the vehicle. Once the angle between the robot-mounted camera and the vehicle is reduced to approximately zero, ARtoolKit calculates the distance between the robot and the vehicle.
(3) The robot moves toward the vehicle and stops after traveling half of the distance between it and the vehicle.
(4) The robot again pivots on its left and right rear wheels until the angle between its front-mounted camera and the vehicle is reduced to approximately zero, after which ARtoolKit again determines the distance between the robot and the vehicle.
  These processes (AR marker detection, pivot turning, and forward motion) are repeated until the distance between the robot and the vehicle is reduced to 0.3 m.
(5) The ultrasonic sensor on the hand then searches for and detects the front bar of the vehicle. Next, the infrared distance sensor on the hand detects the front bar of the vehicle and performs minor position adjustments until the robot hand is able to grasp the front bar (Figure 3). After the bar is grasped, the hand then stops.
(6) The robot moves backward, causing the inclination angle of the vehicle, γ, to increase. The connection position between the robot and the vehicle begins moving up along the front bar. During this process, the robot uses ARtoolKit to monitor both the inclination of the vehicle and the AR marker mounted on its bottom, as shown in Figure 6b.
(7) The robot continues to move backward. When γ is more than 120°, the robot observes the other AR marker mounted on the front of the vehicle, as shown in Figure 6a.
(8) The robot continues to move backward until γ becomes 180°, and then stops. At that point, the vehicle has been recovered from the overturned state.

## 4. Requirements and Statics Analysis during Recovery of Overturned Vehicle

**Figure 9:**
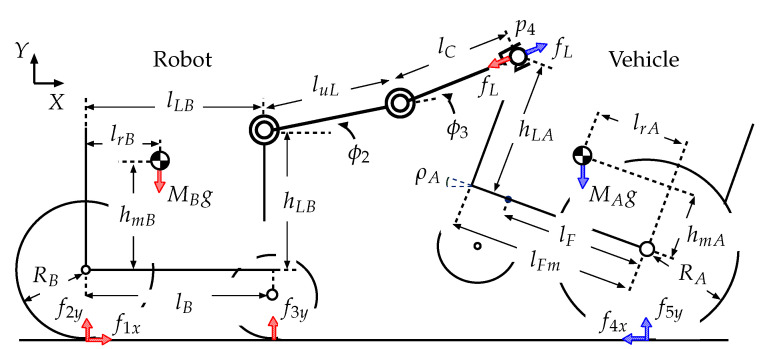
Model of robot and overturned vehicle during recovery process.

In this section, the authors perform a statics analysis to evaluate the possibility of recovering an overturned vehicle. The axles of the robot manipulator are fixed when the robot grasps the vehicle. The robot slowly moves and maintains its balance in the recovery process, which is analyzed by considering statics. Figure 9 shows the state in which the robot rescues the overturned vehicle. Here, $f_{1x}$: driving force for the rear wheels of the robot; $f_{2y}$: normal reaction which affects the rear wheels of the robot; $f_{3y}$: normal reaction which affects the front wheels of the robot; $f_{4x}$: driving force for the rear wheels of the vehicle; $f_{5y}$: normal reaction which affects the rear wheels of the vehicle; $f_{L}$: reaction force from the connected vehicle (or robot); $h_{mB}$: height of the robot center of gravity above the rear wheel axle; $l_{rB}$: distance between the robot center of gravity and the rear wheel axis, $l_{B}$: wheelbase of the robot; $R_{B}$: radius of the robot rear wheels; $M_{B}$: mass of the robot; $l_{LB}$: distance between the shoulder axle of the manipulator and the rear axle; $h_{LB}$: height of the shoulder axle of the manipulator above the rear axle; $l_{uL}$: length of the upper arm link (from the shoulder joint to the elbow joint), $l_{fL}$: length from the elbow joint to the connecting position between the robot and vehicle; $\phi_{2}$: shoulder link angle, $\phi_{3}$: elbow link angle, $p_{4}:{\left[x_{4}\;\;y_{4}\right]}^{T}$: connecting position between the robot and vehicle, $\rho_{A}$: angle of inclination of the vehicle; $h_{mA}$: height of the center of gravity of the robotic vehicle above the rear wheel axle; $l_{rA}$: distance between the center of gravity of the vehicle and the rear wheel axle, $l_{A}$: wheelbase of the vehicle; $R_{A}$: radius of the rear wheels of the vehicle; $M_{A}$: mass of the vehicle; $l_{F}$: distance between the robot hand’s grasping position and the rear axle (in the recovery process, 0.28 m $\leq l_{F}\leq 0.41$ m); $l_{Fm}$: maximum value of $l_{F}$; $h_{LA}$: height of the robot hand’s grasping position; *g*: gravitational acceleration. The dimensions for these parameters are shown in Table A1 and Table A2 in the Appendix A.

### 4.1. Static Requirements to Achieve Recovery of an Overturned Vehicle

The system needs to satisfy conditions *Req.* 1–5 below in order to rescue the vehicle. Here, μ is the coefficient of friction between the driving wheels and the road surface.

Req.1: No part of the robot manipulator contacts the overturned vehicle except the robot hand.
Req.2: The connecting position between the robot and the vehicle is within the range reachable by the robot hand.
Req.3: The driving wheels of the robot do not slip ($\mu >|f_{1x}|/f_{2y}$).
Req.4: The driving wheels of the robotic vehicle do not slip ($\mu >|f_{4x}|/f_{5y}$).
Req.5: The robot does not tip over backward as a result of the force exerted by the tilting vehicle ($f_{3y}>0$).

Req.1 and Req.2 are geometrical requirements. The inclination of the vehicle without interference from the road surface or robot hand is −20° $\leq \rho_{A}\leq$ 100° (Req.1). Considering the position of the center of the gravity of the vehicle, it will not tip over for a range of inclination of 0° $\leq \rho_{A}\leq$ 100°. For this range of inclination, the robot hand is able to grasp the robotic vehicle (Req.2). Thus, the requirements to achieve recovery satisfying Req.3 to Req.5 are analyzed in the range 0° $\leq \rho_{A}\leq$ 100°.

### 4.2. Analysis of Statics to Achieve Vehicle Rescue

When moving statically, the force equilibrium for the robot along the *X* and *Y* axes is given by the following Equation (Figure 9)

(1) $$f_{1x}-f_{L}\cos\phi_{23}=0$$

(2) $$f_{2y}+f_{3y}-M_{B}g-f_{L}\sin\phi_{23}=0$$

where $\phi_{23}=\phi_{2}+\phi_{3}.$

From (1),

(3) $$f_{1x}=f_{L}\cos\phi_{23}$$

From (2),

(4) $$f_{2y}=M_{B}g+f_{L}\sin\phi_{23}-f_{3y}$$

The following equation is obtained from the equilibrium of the moments about the point of contact between the robot rear wheels and the ground.

(5) $$f_{3y}\cdot l_{B}-M_{B}\cdot l_{rB}-x_{4}\cdot f_{L}\sin\phi_{23}+y_{4}\cdot f_{L}\cos\phi_{23}=0$$

where $p_{4}={\left[x_{4}\;\;y_{4}\right]}^{T}$,

(6) $$x_{4}=l_{LB}+l_{uL}\cos\phi_{2}+l_{fL}\cos\phi_{23}$$

(7) $$y_{4}=R_{B}+h_{LB}+l_{uL}\sin\phi_{2}+l_{fL}\sin\phi_{23}$$

From (5),

(8) $$f_{3y}=\frac{M_{B}l_{rB}+f_{L}\left\{x_{4}\sin\phi_{23}-y_{4}\cos\phi_{23}\right\}}{l_{B}}$$

The force equilibrium for the robotic vehicle along the *X* and *Y* axes is given by the following equations.

(9) $$f_{L}\cos\phi_{23}-f_{4x}=0$$

(10) $$f_{L}\sin\phi_{23}-M_{A}g+f_{5y}=0$$

From (9),

(11) $$f_{4x}=f_{L}\cos\phi_{23}$$

From (10),

(12) $$f_{5y}=M_{A}g-f_{L}\sin\phi_{23}$$

**Figure 10:**
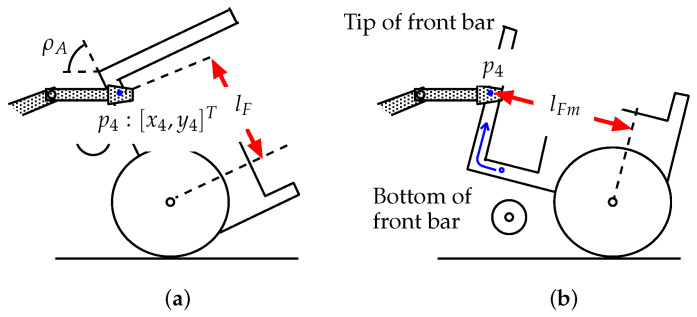
Connecting position and inclination of the vehicle: (**a**) situation where the hand is lower than the tip of the front bar ($y_{4}<R_{A}+l_{Fm}\sin\rho_{A}$); (**b**) situation where the hand is higher than the tip of the front bar ($y_{4}\geq R_{A}+l_{Fm}\sin\rho_{A}$).

When the hand position is lower than the tip of the front bar ($y_{4}<R_{A}+l_{Fm}\sin\rho_{A}$, thus 49.74° $<\rho_{A}<$ 100°, Figure 10a), the robot hand grasps the bottom part of the front bar of the overturned vehicle (Figure 5). In this case, the equilibrium of moment of the vehicle about the point of contact between the rear wheels and the ground is given below.

(13) $$\begin{array}{c} \left(l_{rA}\cos\rho_{A}-h_{mA}\sin\rho_{A}\right)M_{A}g-f_{L}\cos\phi_{23}\cdot \left(R_{A}+l_{F}\sin\rho_{A}\right)\\ -f_{L}\sin\phi_{23}\cdot l_{F}\cos\rho_{A}=0 \end{array}$$

where $l_{F}$ is a variable that depends on the inclination of the vehicle. In this case, the reaction force exerted by the connected vehicle (or robot), $f_{L}$, is expressed by the following Equation (13).

(14) $$\begin{array}{c} f_{L}=\frac{\left(l_{rA}\cos\rho_{A}-h_{mA}\sin\rho_{A}\right)M_{A}g}{\left(R_{A}+l_{F}\sin\rho_{A}\right)\cos\phi_{23}+l_{F}\cos\rho_{A}\sin\phi_{23}} \end{array}$$

where 49.74° $<\rho_{A}<$ 100°.

The height of the hand, $y_{4}$, is expressed by the following Equation (Figure 9).

(15) $$y_{4}=R_{A}+l_{F}\sin\rho_{A}$$

The following equation is obtained from (7) and (15).

(16) $$l_{F}=\frac{R_{B}+h_{LB}+l_{uL}\sin\phi_{2}+l_{fL}\sin\phi_{23}-R_{A}}{\sin\rho_{A}}$$

When the hand position becomes higher than the vehicle’s front bar ($y_{4}\geq R_{A}+l_{Fm}\sin\rho_{A}$, thus 0 ≤ 49.74°), Figure 10b), the hand grasps the tip of the front bar. The equilibrium vehicle moment about the point of contact between the rear wheels and the ground is then given below.

(17) $$\begin{array}{c} \left(l_{rA}\cos\rho_{A}-h_{mA}\sin\rho_{A}\right)M_{A}g-f_{L}\cos\phi_{23}\cdot \left(R_{A}+l_{Fm}\sin\rho_{A}+h_{LA}\cos\rho_{A}\right)\\ -f_{L}\sin\phi_{23}\cdot \left(l_{Fm}\cos\rho_{A}-h_{LA}\sin\rho_{A}\right)=0 \end{array}$$

When $y_{4}\geq R_{A}+l_{Fm}\sin\rho_{A}$ (thus, 0 ≤ 49.74°), $l_{F}$ is constant.

(18) $$l_{F}=l_{Fm}\left(\;=0.41\;m\right).$$

The following equation is obtained geometrically (Figure 9).

(19) $$R_{A}+l_{Fm}\sin\rho_{A}+h_{LA}\cos\rho_{A}=R_{B}+h_{LB}+l_{uL}\sin\phi_{2}+l_{fL}\sin\phi_{23}$$

From (19),

(20) $$h_{LA}=\frac{R_{B}-R_{A}+h_{LB}+l_{uL}\sin\phi_{2}+l_{fL}\sin\phi_{23}-l_{Fm}\sin\rho_{A}}{\cos\rho_{A}}.$$

In this case, the reaction force exerted by the connected vehicle (or robot), $f_{L}$, is expressed by following Equation (17).

(21) $$\begin{array}{c} f_{L}=\frac{\left(l_{rA}\cos\rho_{A}-h_{mA}\sin\rho_{A}\right)M_{A}g}{\left(R_{A}+l_{Fm}\sin\rho_{A}+h_{LA}\cos\rho_{A}\right)\cos\phi_{23}+\left(l_{Fm}\cos\rho_{A}-h_{LA}\sin\rho_{A}\right)\sin\phi_{23}} \end{array}$$

where $y_{4}\geq R_{A}+l_{F}\sin\rho_{A}$

The relation $|f_{1x}|/f_{2y}<\mu$ required by Req.3 can be evaluated from (8)–(18), (14) and (21), and the relation $|f_{4x}|/f_{5y}<\mu$ required by Req.4 can be evaluated from (11)–(14) and (21).

**Figure 11:**
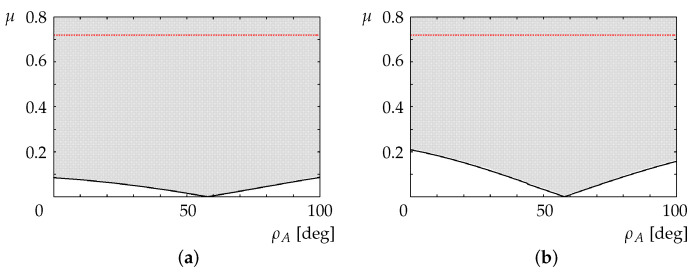
Inclination of overturned vehicle and friction coefficient for avoiding slippage of (**a**) robot driving wheels and (**b**) vehicle driving wheels.

**Figure 12:**
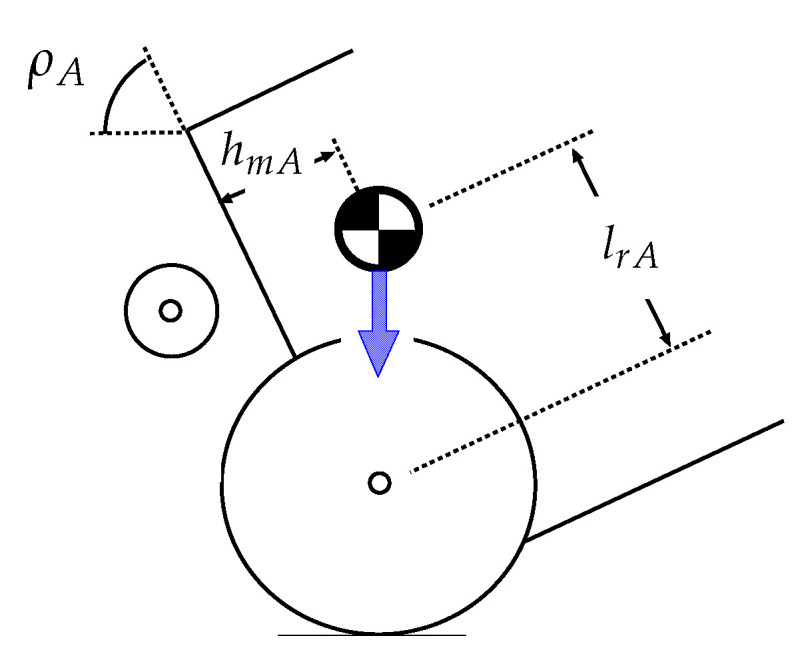
Situation in which the horizontal position of the center of gravity of the vehicle is above the contact position of the rear wheels.

The shaded area in Figure 11a shows the relationship between the inclination of the overturned vehicle and the coefficient of friction between the robot rear wheels and the road surface required to avoid slippage of the robot driving wheels (Req.3). Similarly, the shaded area in Figure 11b shows the relationship between the inclination of the vehicle and the coefficient of friction that ensures no slippage of the vehicle wheels (Req.4). The lowest points in Figure 11a,b occur at $\rho_{A}=57.86$, when the horizontal position of the center of gravity of the vehicle is above the contact position of the rear wheels (Figure 12).

Experiments were conducted for a coefficient of friction of μ=0.72 (see Section 6), for which the driving wheels of the robot and the vehicle could generate enough torque to avoid tipping over.

**Figure 13:**
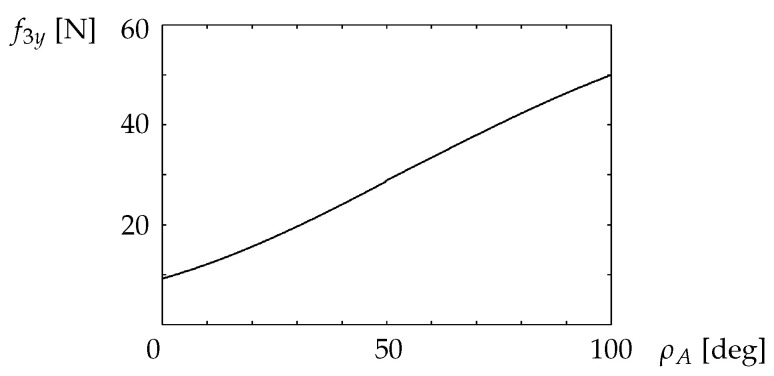
Relationship between the coefficient of friction and the inclination of the overturned vehicle that prevents the robot from tipping over backward.

The relation $f_{3y}>0$ required by Req.5 is evaluated using (8), (14), and (21). Figure 13 shows the relationship between the inclination of the vehicle and the normal force on the robot front wheels, $f_{3y}$ (Req.5). For 0 $\leq \rho_{A}\leq 100$°, the front wheels of the robot are not lifted, and the robot does not overturn due to the force exerted by the vehicle.

In theory, if the geometric requirements Req.1 and Req.2 are satisfied, the robot can successfully perform the recovery operation because Req.3 to Req.5 can also be satisfied.

## 5. Theoretical Analysis of the Distance between the Robot Hand and the Overturned Vehicle

**Figure 14:**
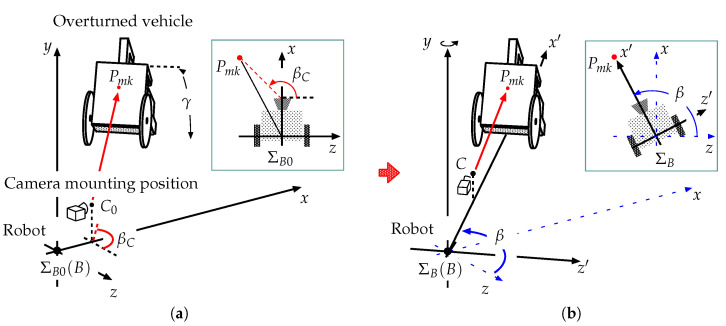
Model showing robot and overturned vehicle: (**a**) relative angle and (**b**) robot oriented towards vehicle.

Figure 14a shows the initial status of the robot and the overturned vehicle. The basic robot coordinate system is denoted by $\Sigma_{B0}$, where point *B* is the origin. Here, point *B* is the center between the two floor surface contact positions for the left and right rear wheels. In this figure, *x* indicates the roll axle for the initial direction of the robot, *y* indicates the yaw axle, and *z* indicates the pitch axle. $C_{0}$ indicates the camera mounting position on the robot in $\Sigma_{B0}$.

β is the angle formed by the *x* axle and the line from point *B* to point *M* (AR marker position) on the xz plane. Similarly, $\beta_{C}$ is the relative angle, which is formed by the *x* axle and the line from $C_{0}$ to *M* on the xz plane.

When the robot turns toward the vehicle, $\Sigma_{B0}$, *x*, and *z* change to $\Sigma_{B}$, $x^{\prime }$, and $z^{\prime }$, respectively (see Figure 14b). The camera position on the robot, $C_{0}$, changes to *C*.

**Figure 15:**
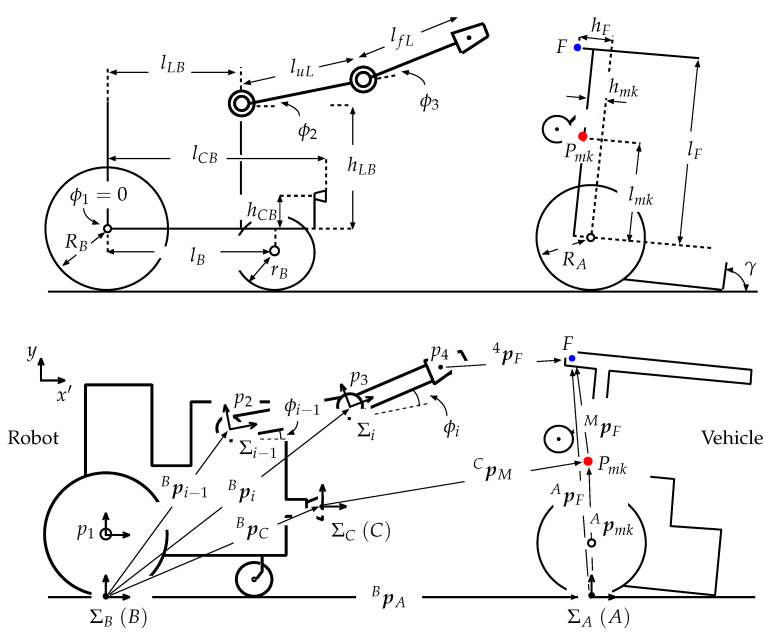
Model showing overturned vehicle recovery.

Figure 15 shows the situation following a pivot turn when the robot is oriented toward the vehicle. The local coordinate system for the camera mounted on the robot is denoted as $\Sigma_{C}$, which is always parallel to $\Sigma_{B}$. The position vector for the camera in $\Sigma_{B}$ is expressed as ${}^{B}p_{C}$.

The camera position, ${}^{B}p_{C}$, is given by (Figure 2),

(22) $${}^{B}p_{C}:\left[\begin{array}{c} x_{C}\\ y_{C}\\ z_{C} \end{array}\right]=\left[\begin{array}{c} l_{CB}\\ R_{B}+h_{CB}\\ 0 \end{array}\right]$$

where $\phi_{i}$ is the angle of the local coordinate system ($\Sigma_{i}$), $\Sigma_{i}$ is formed by $\Sigma_{i-1}$. In this system, both the inclination of the robot and $\phi_{1}$ are zero. The position vectors for these joints in $\Sigma_{B}$ are expressed as ${}^{B}p_{i}={\left[x_{i}\;\;y_{i}\;\;z_{i}\right]}^{T}\;\;\;\left(i=1-4\right)$, where the robot hand position ${}^{B}p_{4}$ (Figure 2) is given by:

(23) $${}^{B}p_{4}\left[\begin{array}{c} x_{4}\\ y_{4}\\ z_{4} \end{array}\right]=\left[\begin{array}{c} l_{fL}\cos\phi_{23}+l_{uL}\cos\phi_{2}+l_{LB}\\ l_{fL}\sin\phi_{23}+l_{uL}\sin\phi_{2}+h_{LB}+R_{B}\\ 0 \end{array}\right]$$

where $\phi_{23}=\phi_{2}+\phi_{3}$. 

As shown in Figure 15, the inclination angle of the overturned vehicle is γ, and its local coordinate system is denoted as $\Sigma_{A}$. Here, point A is the center between the two surface contact positions for the left and right rear wheels, point *M* is the position of the AR marker mounted on the vehicle bottom, and point *F* is the position where the robot grasps the vehicle. ${}^{A}p_{M}={{[}^{A}x_{M}\;{\;}^{A}y_{M}\;{\;}^{A}z_{M}]}^{T}$ is the AR marker position vector in $\Sigma_{A}$, given by:

(24) $${}^{A}p_{M}:\left[\begin{array}{c} {}^{A}x_{M}\\ {}^{A}y_{M}\\ {}^{A}z_{M} \end{array}\right]=\left[\begin{array}{c} l_{M}\cos\gamma +h_{M}\sin\gamma \\ l_{M}\sin\gamma -h_{M}\cos\gamma +R_{A}\\ 0 \end{array}\right]$$

${}^{A}p_{F}$ is the position vector for point *F* in $\Sigma_{A}$, expressed as:

(25) $${}^{A}p_{F}:\left[\begin{array}{c} {}^{A}x_{F}\\ {}^{A}y_{F}\\ {}^{A}z_{F} \end{array}\right]=\left[\begin{array}{c} l_{F}\cos\gamma +h_{F}\sin\gamma \\ l_{F}\sin\gamma -h_{F}\cos\gamma +R_{A}\\ 0 \end{array}\right]$$

The position vector for the AR marker in $\Sigma_{C}$ is ${}^{C}p_{M}$, given by

(26) $${}^{C}p_{M}=\left[\begin{array}{c} {}^{C}x_{M}\\ {}^{C}y_{M}\\ {}^{C}z_{M} \end{array}\right]$$

${}^{C}p_{M}={{[}^{C}x_{M}\;{\;}^{C}y_{M}\;{\;}^{C}z_{M}]}^{T}$ is obtained using ARToolKit.

When the robot is oriented towards the vehicle, ${}^{C}z_{M}=0$. In this case,

(27) $${}^{C}p_{M}=\left[\begin{array}{c} {}^{C}x_{M}\\ {}^{C}y_{M}\\ 0 \end{array}\right]$$

Geometrically, the authors obtained Equation (28), as shown in Figure 14a:

(28) $$\tan\beta_{C}=\frac{{}^{C}z_{M}}{{}^{C}x_{M}}$$

Using ARtoolKit, the robot conducts a pivot turn until the value of $\beta_{C}$ (Figure 14a) becomes approximately zero, and it is oriented towards the vehicle. In this case, the position vector *A* in $\Sigma_{B}$ is expressed as (Figure 15):

(29) $${}^{B}p_{A}={}^{B}p_{C}+{}^{C}p_{M}-{}^{A}p_{M}$$

From Equations (22), (24) and (27),

(30) $${}^{B}p_{A}=\left[\begin{array}{c} l_{CB}{+}^{C}x_{M}-\left(l_{M}\cos\gamma +h_{M}\sin\gamma \right)\\ R_{B}+h_{CB}{+}^{C}y_{M}-\left(l_{M}\sin\gamma -h_{M}\cos\gamma \right)\\ 0 \end{array}\right]$$

The vector from the robot hand to the grasping position is ${}^{4}p_{F}={{[}^{4}x_{F}\;{\;}^{4}y_{F}\;{\;}^{4}z_{F}]}^{T}$,

(31) $${}^{4}p_{F}=-{}^{B}p_{4}+{}^{B}p_{A}+{}^{A}p_{F}$$

From Equations (23), (25) and (30),

(32) $${}^{4}p_{F}:\left[\begin{array}{c} {}^{4}x_{F}\\ {}^{4}y_{F}\\ {}^{4}z_{F} \end{array}\right]=\left[\begin{array}{c} {}^{C}x_{M}-H_{x}+l_{C}+\left(l_{F}-l_{M}\right)\cos\gamma -\left(h_{M}-h_{F}\right)\sin\gamma \\ {}^{C}y_{M}-H_{y}+h_{C}+\left(l_{F}-l_{M}\right)\sin\gamma +\left(h_{M}-h_{F}\right)\cos\gamma +R_{A}\\ 0 \end{array}\right]$$

where $H_{x}=l_{fL}\cos\phi_{23}+l_{uL}\cos\phi_{2}+l_{LB}$, $H_{y}=l_{fL}\sin\phi_{23}+l_{uL}\sin\phi_{2}+h_{LB}$. 

When the robot identifies the grasping position on the vehicle, it can set the hand at the same height as point *F* on the vehicle. In this case, ${}^{4}y_{F}=0$ in Equation (32), and the distance between the hand and point *F* is expressed as

(33) $${|}^{4}p_{F}{\left|=\right|}^{4}x_{F}|=\sqrt{{{\{}^{C}x_{M}-H_{x}+l_{CB}+\left(l_{F}-l_{M}\right)\cos\gamma -\left(h_{M}-h_{F}\right)\sin\gamma \}}^{2}}$$

The robot moves in order to reduce ${|}^{4}p_{F}|$ to zero using its sensors and ARtoolKit. At that point, the robot can grasp the overturned vehicle.

## 6. Experiment

### 6.1. Conditions under Which the Robot Can Rescue the Vehicle

An experiment was carried out in an environment with a surface friction coefficient of μ=0.68. The robot and overturned vehicle were placed on the same floor within the National Institute of Technology, Toyama College. The maximum movement speed of the robot was set at 3.6 km/h.

The authors began by conducting experiments to ensure that the AR markers could be detected using the robot-mounted camera. From the results obtained, the authors found that ARtoolkit sometimes reacted incorrectly in situations where the camera captured furniture or posters with a lattice-like pattern. In those situations, the robot could not be properly controlled. Accordingly, the external environment in which the robot and vehicle experiments were conducted had a white wall and was further partitioned from the rest of the room by suspending a piece of white cloth between the robot and any potentially distracting objects.

**Figure 16:**
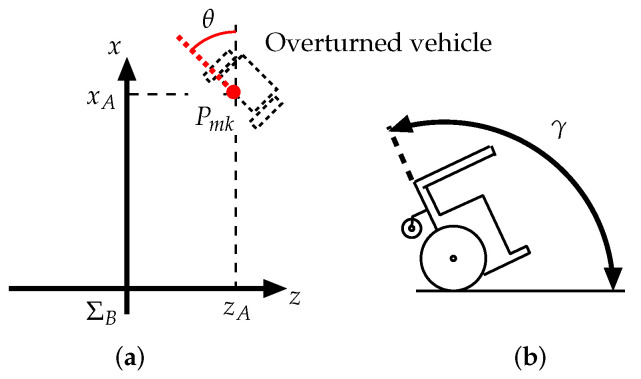
Position and angle of overturned vehicle: (**a**) position and (**b**) angle.

The authors then determined that the camera could detect the AR markers mounted on the overturned vehicle at distances of up to 1.5 m, and that when the robot and the vehicle were separated by less than 1.5 m, the camera was able to capture the AR marker position for −0.4 m $\leq z_{A}\leq$ 0.4 m, as shown in Figure 16. Furthermore, when the relative angle between the AR marker and the camera, θ, was −35° ≤θ≤ 35°, the camera could detect the AR marker. The body angle of the overturned vehicle, γ, could be detected for 78° ≤γ≤ 136°.

The recovery experiments were carried out from the overturning point shown in Figure 16. Here, $x_{A}=1.5$ m, $z_{A}=0.4$ m, the relative angle between the AR marker and the camera was θ=90°, and the first inclination angle of the vehicle was set at γ=90°. The robot detected the AR marker mounted on the vehicle bottom within γ=90° ≤γ≤ 120°, as shown in Figure 6b, and when the inclination angle of the vehicle was over 120°, the robot detected the AR marker mounted on the vehicle front, as shown in Figure 6 a.

We assumed that the robot had already determined the correct position to grasp the vehicle in order to achieve successful recovery ($h_{F}=20$ mm, $l_{F}=320$ mm). We also assumed that the driving wheels of the robot and vehicle would not slip, and that the robot could generate sufficient force to pull and support the overturned vehicle during the recovery process.

### 6.2. Experimental Test of Recovery of Overturned Wheeled Vehicle

**Figure 17:**
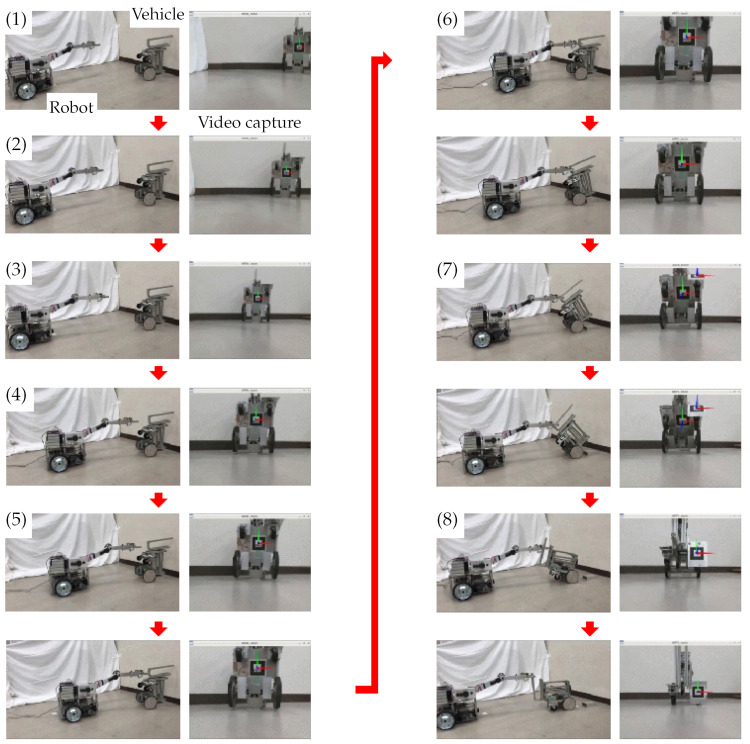
Experiment. The states shown in Figure 17 correspond to (1)–(8) described earlier.

The states shown in Figure 17 correspond to (1)–(8) described earlier. The left panels show movements of the robot and vehicle, and the right panels show video images captured by the camera mounted on the robot. The arrows on the right side indicate that ARtoolkit was able to detect the AR markers.

The camera on the robot captured video images of the vehicle at the screen center. Then, based on its perception of the AR marker mounted on the vehicle bottom, the robot repeated its pivot turn to correct its orientation relative to the vehicle. The robot then traveled half of the distance between it and the vehicle, stopped, and repeated the AR marker detection, pivot turn, and forward motion processes until the robot hand was able to grasp the front bar of the vehicle.

Once the front bar was grasped, the robot began moving backward, exerting a pulling force on the overturned vehicle and causing γ to increase. Up to that point, the robot’s actions were based on its detection of the AR marker mounted on the vehicle bottom. However, once γ exceeded 120°, the robot detected the vehicle’s front-mounted AR marker instead of the bottom AR marker. The robot continued moving backward until γ= 180°, at which time it stopped. At that point, the vehicle was back on its wheels, and the recovery process was complete.

As described above, it was necessary to modify the external environment in which the robot and vehicle operated during the experiment to make it suitable for ARtoolKit use. This required removing or hiding objects with lattice-like patterns. Once this was accomplished, the robot was able to work stably, and successfully recovered the overturned vehicle.

## 7. Conclusions and Future Work

This paper described potential recovery tactics to use when employing a mobile robot to recover a vehicle that had fallen over backward and showed how such a robot system could be constructed. A theoretical analysis clarified the relationship between the vehicle’s angle and the coefficient of friction required to recover an overturned vehicle. Determining the distance between the robot and the overturned vehicle made it possible for the robot to grasp the vehicle and return it to an upright position.

Since the results showed that ARtoolkit sometimes reacted incorrectly when the robot’s camera captured furniture or posters with lattice-like patterns, such items were removed or covered, and the experiments in this study were conducted in an environment that allowed ARtoolKit to correctly perceive the overturned vehicle. The robot was able to work stably and recover the overturned vehicle. Despite this restriction, the experimental results obtained confirmed the effectiveness of the proposed method. Therefore, it is thought that by further developing this system, it would be possible to establish a new technology that would allow vehicles to be easily recovered when an accident occurs. This would be particularly useful if such vehicles became incapacitated in areas that are inaccessible to humans. In the future, the authors will conduct experiments under various other environments and validate sensor outputs during the recovery process in order to improve the robot’s perception capabilities.

The authors will also construct a judgment system for determining the most suitable grasping positions when recovering an overturned vehicle and will study other recovery methods to be used when a vehicle overturns or becomes stuck due to driving wheel slippage when traversing rough terrain. In addition, the authors will construct a manipulator with a hand mechanism that is suitable for rescuing an overturned vehicle. The authors will study appropriate rescue methods for various vehicle tipping conditions, such as rolling.

## Data Availability

Not applicable.

## Appendix A. Robot and Vehicle Specifications

**Table A1:** Robot specifications.

Overall length	490 mm
Overall height	250 mm
Body width	430 mm
Radius of front wheels ($r_{Bf}$)	12.5 mm
Radius of rear wheels ($R_{B}$)	90 mm
Wheelbase $\left(l_{B}\right)$	225 mm
Distance between the shoulder axle and the rear axle $\left(l_{LB}\right)$	150 mm
Height of the shoulder axle above the rear axle $\left(h_{LB}\right)$	190 mm
Camera position $\left(l_{CB}\right)$	300 mm
Camera height $\left(h_{CB}\right)$	−28 mm
Length of upper link (between the shoulder joint and elbow joint) ($l_{uL}$)	330 mm
Length of forearm link (between the elbow joint and hand grasping position) ($l_{fL}$)	370 mm
Height of center of gravity ($h_{mB}$)	122 mm
Distance of center of gravity $\left(l_{rB}\right)$	195 mm
Mass $\left(M_{B}\right)$	17.0 kg
Driving mechanism	Individually driven wheels

**Table A2:** Overturned vehicle specifications.

Overall length	450 mm
Overall height	355 mm
Body width	250 mm
Radius of front wheels $\left(r_{A}\right)$	35 mm
Radius of rear wheels $\left(R_{A}\right)$	80 mm
Wheelbase $\left(l_{A}\right)$	190 mm
Position of center of gravity $\left(l_{rA}\right)$	78 mm
Height of center of gravity $\left(h_{mA}\right)$	49 mm
Target position for connection from the rear axle $\left(l_{F}\right)$	285–410 mm
Position of front bar ($l_{Fm}$: maximum value of $l_{F}$)	410 mm
Target height for connection from the rear axle $\left(h_{F}\right)$	20 mm
Mass $\left(M_{A}\right)$	6.5 kg
Driving mechanism	Individually driven wheels
